# Response to the Letter to Editor:” Is Micronucleus Assay Suitable for Cytogenetic Biomonitoring the Different Ways to Smoke? ”

**DOI:** 10.30699/ijp.2020.127517.2390

**Published:** 2020-08-20

**Authors:** Noushin Jalayer Naderi

**Affiliations:** *Department of Oral and Maxillofacial Pathology, Faculty of Dentistry, Shahed University, Tehran, Iran*

Dear Editor,

I was very pleased to find that the article” Micronucleus Assay of Buccal Mucosa Cells in Waterpipe (Hookah) Smokers: A Cytologic Study “(1) has attracted the attention of some readers and has created a few points for them. In my opinion, these points are good bases to overcome some concerns regarding micronucleus assay.

Regarding the first question, keratohyalin granules are round spots, irregular spherical globules associated with tonofibrils that dispersed in intermediate layer of stratified squamous epithelium. Feulgen which is a DNA-specific stain, has shown that the DNA was not a constituent element of keratohyalin granules. Actually, keratohyalin granules are allied to the stratum granulosum in ortho-keratinization process and keratin formation (2,3). Due to the histopathologic nature and appearance, failing to distinguish keratohyalin granules from micronucleus by a calibrated person is an unjustifiable and frustrating mistake especially in Feulgen stained slides. Careful attention to the Figure used in the article of interest (1) shows the micronuclei have the same characteristic mentioned as inclusion criteria in Material and Methods. Micronuclei are smaller than nucleus and are in a short distance from it. A decade of experiences in study of micronuclei and reported findings have shown the reviewers are interested in saturated chromatic images. For this reason, a yellow filter was used for imaging the slides. *Note the background of the image*, it is brighter yellow than usual. In terms of experience, I always capture an unfiltered image of the samples at the same position as filtered images, so I will share it in this text. In this unfiltered image (Figure 1), the color of the micronuclei is the same as the nucleus. The green-blue color of the cytoplasm is clearly visible. Figure 1 obviously shows the sample was stained with Feulgen.

**Fig. 1:**
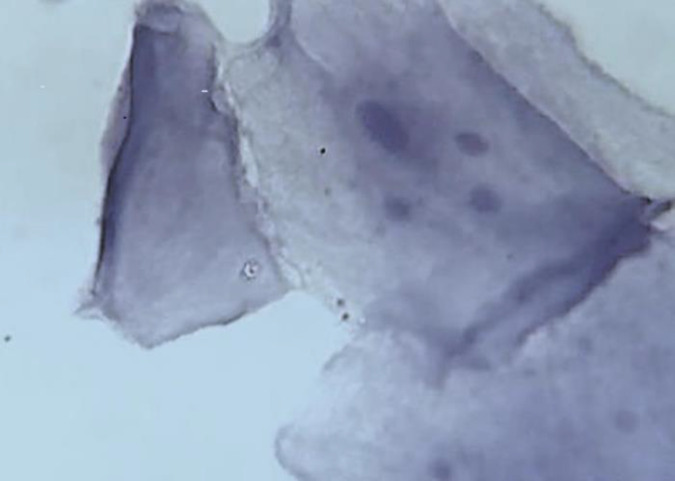
Micronuclei in buccal mucosa smear of waterpipe smoker *(× 1000, Feulgen staining)*

The second question was addressed investigation of total number of the cells. Our reference to conduct the study was the study conducted by El-Setouhy, *et al.* (2008) (4)*. *Based on it, a total of 1000 cells were evaluated. Counted cells were varied from 500 (5), 1000 (6), 2000 (7) and even to 3000 (8) cells per subject in different studies. Calculating the 4000 cells per subject to overcome the confidence interval in micronucleus assay has been recommended in a review study (9). So far, no clinical study has compared the difference between scoring of 1000 cells Vs >2000 cells in the results of micronucleus assay. Whatever the case may be, in any count of buccal mucosa cells, number of micronucleus was higher in people who were exposed to chemicals. Consequently, based on available studies, it is difficult to judge whether the count of > 2000 cells would be preferred to 1000 cells. Thomas *et al.* in 2009 (10) established a protocol on micronucleus assay performance, but it seems that does not run globally. A review of the literature shows most researchers still test 1000 buccal mucosal cells to detect the quantitative changes of micronuclei. Basically, the micronucleus assay requires a validated protocol which comprises different aspects of background information such as demographic variables and inclusion /exclusion criteria that is followed by all researchers.

The third question addressed the data presentation. The mean number of micronuclei in buccal mucosa of non-smokers were 1.68±0.35 (1). Based on Ceppi *et al.* in 2010 (9) and Bonassi *et al.* in 2011(11), frequency of micronuclei in healthy individuals were 0.70–1.72 and 0.3–1.7%, respectively.

 Accordingly, the average of 1.68 is in the range of previous studies. It should be emphasized that the number of micronuclei in buccal mucosa depends on some factors such as personal lifestyle and history of exposure to chemicals (11). Basically, it is more scientific to compare the micronucleus count in buccal mucosa in healthy and subject individuals in a same community.

I also agree with the authors that the arrangement of comments by researchers who work in the field of micronucleus assay can resolve the discrepancies in the method of work and help to improve a standard method. Biomonitoring of buccal mucosa cells is a noninvasive, and useful method to detect the genotoxic and cytotoxic effects. A standard protocol can make this known method as an efficient screening method.

## Conflict of Interest

**The author declared no conflict of interest.**

## References

[1] DehghanNezhad M, Jalayer Naderi N, Semyari H (2020). Micronucleus Assay of Buccal Mucosa Cells in Waterpipe (Hookah) Smokers: A Cytologic Study. Iran J Pathol.

[2] Nanci A (2018). Ten Cate's Oral Histology; Development, Structure, and Function.

[3] Holbrook KA (1989 ). Biologic structure and function: perspectives on morphologic approaches to the study of the granular layer keratinocyte. J Invest Dermatol.

[4] El-Setouhy M, Loffredo CA, Radwan G, Rahman RA, Mahfouz E, Israel E, Mohamed MK, Ayyad SB (2008 ). Genotoxic effects of waterpipe smoking on the buccal mucosa cells. Mut Res/Genetic Toxicology and Environmental Mutagenesis.

[5] Farhadi S, Mohamadi M, Mohamadi M (2017). Repair Index in Examination of Nuclear Changes in the Buccal Mucosa of Smokers: A Useful Method for Screening of Oral Cancer. Asian Pac J Cancer Prev.

[6] Grover S, Mujib A, Jahagirdar A, Telagi N, Kulkarni P (2012). A comparative study for selectivity of micronuclei in oral exfoliated epithelial cells. J Cytol.

[7] Konopacka M (2003). Effect of smoking and aging on micronucleus frequencies in human exfoliated buccal cells. Neoplasma.

[8] Martino-Roth MG, Viégas J, Amaral M, Oliveira L, Ferreira FLS, Erdtmann B (2002). Evaluation of genotoxicity through micronuclei test in workers of car and battery repair garages. Genet Mol Biol.

[9] Ceppi M, Biasotti B, Fenech M, Bonassi S (2010). Human population studies with the exfoliated buccal micronucleus assay: statistical and epidemiological issues. Mutat Res.

[10] Thomas P, Holland N, Bolognesi C, KirschVolders M, Bonassi S, Zeiger E (2009). Buccal micronucleus cytome assay. Nat Protoc.

[11] Bonassi S, Coskun E, Ceppi M, Lando C, Bolognesi C, Burgaz S (2011). The HUman MicroNucleus project on eXfoLiated buccal cells (HUMNXL): The role of life-style, host factors, occupational exposures, health status, and assay protocol. Mutat Res.

